# Recurrent Acute Pancreatitis and Superior Mesenteric Venous Thrombosis – Cause or Course

**DOI:** 10.7759/cureus.18558

**Published:** 2021-10-07

**Authors:** Okelue E Okobi, Bryan Dawkins, Janaki Saoji, Kevin Nyabera, Daphne Metellus, Vera Hapshy, Azeberoje Osueni, Ishan A Gunawardene, Jennifer Dorcé-Medard

**Affiliations:** 1 Family Medicine, Lakeside Medical Center, Belle Glade, USA; 2 Nephrology, Brookdale University Hospital Medical Center, New York, USA

**Keywords:** acute pancreatitis complications, recurrent, acute, abdominal pain, smvt, superior mesenteric vein thrombosis, pancreatitis

## Abstract

The management of pancreatitis can be daunting, especially when associated with other comorbidities. These complexities in management are conflicting in the presence of comorbidities with a similar presentation, such as abdominal pain. Acute pancreatitis (AP) has been associated with mesenteric thrombosis but less commonly with superior mesenteric vein thrombosis (SMVT) as a causal or complicating dilemma. This case report describes the careful intrigues and overlaps in presentation. Furthermore, this paper presents a dilemma in that contrast-enhanced computed tomography (CT) may not be recommended in the early stage of diagnosis of AP according to the 2013 American College of Gastroenterology (ACG) guideline, but SMVT, which can be fatal, sometimes, complicates AP, and contrast-enhanced CT is important in its diagnosis. This paper attempts to address this dilemma. Managing these two potentially fatal pathologies requires promptness and thoughtfulness in averting a deadly outcome. Because SMVT is fatal, in this paper, we reiterate the use of contrast-enhanced CT in the early stages of the management of AP. Fatal complications from AP should not be missed. Although contrast-enhanced CT is not recommended in the early stages of diagnosis of AP in the ACG guideline, fatal complications such as SMVT can be avoided.

## Introduction

Pancreatitis is one of the common gastrointestinal diseases characterized by inflammation of the pancreas, leading to a systemic inflammatory response [1]. Pancreatitis can result in many vascular complications, both arterial and venous [2]. Venous complications usually manifest as thrombosis of the superior mesenteric or portal vein. It is known that pancreatitis occurring with isolated superior mesenteric vein thrombosis (SMVT) alone is generally associated with a neoplasm or intra-abdominal sepsis [2]. Both entities' diagnostic and treatment guidelines share disparities and similarities that may pose a dilemma during management. In this case presentation, we report a case of recurrent acute pancreatitis (AP) with associated SMVT and its features resembling previously similar written accounts. Furthermore, this paper presents a dilemma in that contrast-enhanced computed tomography (CT) may not be recommended in the early stage of diagnosis of AP according to the 2013 American College of Gastroenterology (ACG) guideline, but SMVT, which can be fatal, sometimes, complicates AP, and contrast-enhanced CT is important in the diagnosis of an SMVT. This paper attempts to address this dilemma. Managing these two potentially fatal pathologies requires promptness and thoughtfulness in averting a fatal outcome. Because SMVT is fatal, we reiterate the use of contrast-enhanced CT in the early stages of management of AP. Fatal complications from AP should not be missed. Although contrast-enhanced CT is not recommended in the early stages of the diagnosis of AP in the ACG guideline, fatal complications such as SMVT can be avoided.

## Case presentation

The patient is a middle-aged male with a past medical history of pancreatitis and alcohol use disorder who was admitted for epigastric pain, described as a stabbing, non-colicky pain that radiated to the back, relieved by position (knee-to-chest-position) with no aggravating factor. He reported that the pain started gradually about 24 hours before presentation after taking 10 glasses of vodka and gin two days before presentation. The patient said that the pain was the worst pain of his life and rated it 10/10 in severity. He denied fever, chills, nausea, and vomiting.

The patient reported a history of recurrent admissions for laboratory and CT diagnosis of AP. He also denied any personal or family history of coagulation or bleeding disorders. A review of other systems did not reveal any contributory supporting findings.

On physical examination, abdomen was flat, moved with respiration, soft, tender epigastric region, no palpable masses, no guarding, no rebound. Bowel sounds were normal; other systemic physical examinations were essentially normal. Vital signs and laboratory values are tabulated in Table 1.

**Table 1:** Vital signs and laboratory values

Test	Value	Reference range	Unit
Blood pressure	158/78	<120/80	mmHg
Heart rate	79	60-99	Beats/minute
Oxygen saturation	100	>95	Percent at room air
Temperature	36.7	36.1-37.2 (oral)	°Celsius
Lipase	3942	65-230	U/l
Ethanol	<3	0-10	mg/dl
Hemoglobin	14.4	14-17.5	g/dl
White blood count	6600	4500-11,000	Cells per microliter
Alkaline phosphatase	105	46-116	U/l
Aspartate aminotransferase	29	15-37	U/l
Alanine aminotransferase	20	12-78	U/l
Total bilirubin	0.6	0.0-1.0	mg/dl
Cholesterol	169	0-200	mg/dl
Triglyceride	67	30-200	mg/dl
High-density lipoprotein	53	40-60	mg/dl
Low-density lipoprotein	103	<100	mg/dl
Sodium	138	136-145	mg/dl
Potassium	4.9	3.5-5.1	mg/dl
Calcium	9.8	8-2-10.2	mg/dl
Creatinine	0.7	0.5-1.2	mg/dl
Glomerular filtration rate	153	>60	ml/min/1.73 m^2^
Chest X-ray	Normal		
Electrocardiogram	Normal sinus rhythm		

Hospital course

Given the previous history of AP and currently elevated lipase levels, the patient was managed for recurrent AP. He was placed on nil per os (NPO), given intravenous fluids and optimum pain medications. Thiamine, dextrose saline, and folic acid were also initiated. On hospital day 2, the patient received further opioids and other possible combinations of analgesic doses. Despite efforts toward effective pain management, his pain persisted and got out of proportion. Further investigation with imaging was required to determine other possible etiology for his abdominal pain. 

Radiological findings

**Figure 1:**
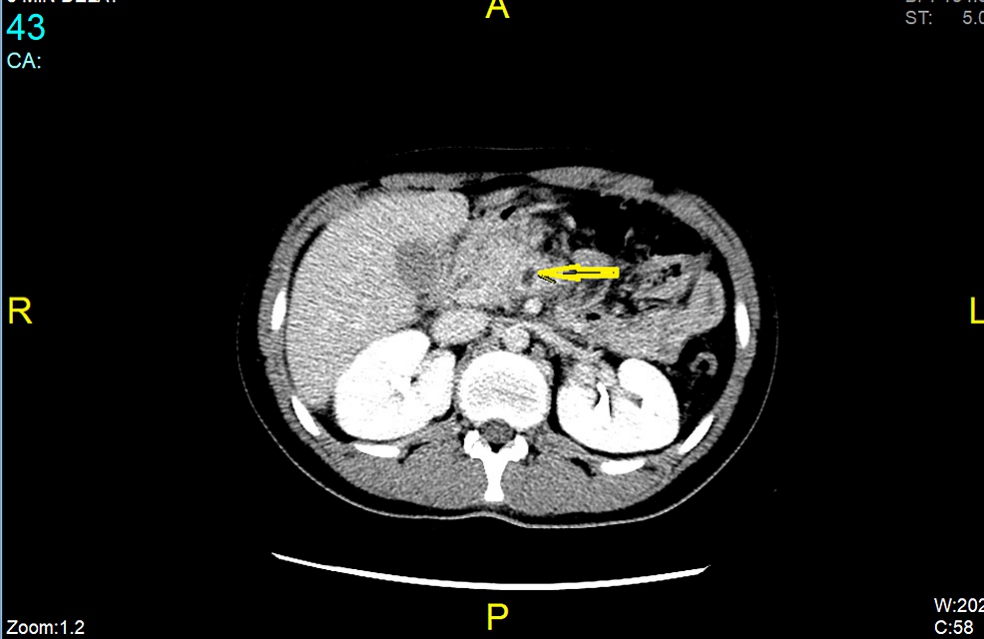
Superior mesenteric vein with unenhancing hypodense filling defect

**Figure 2:**
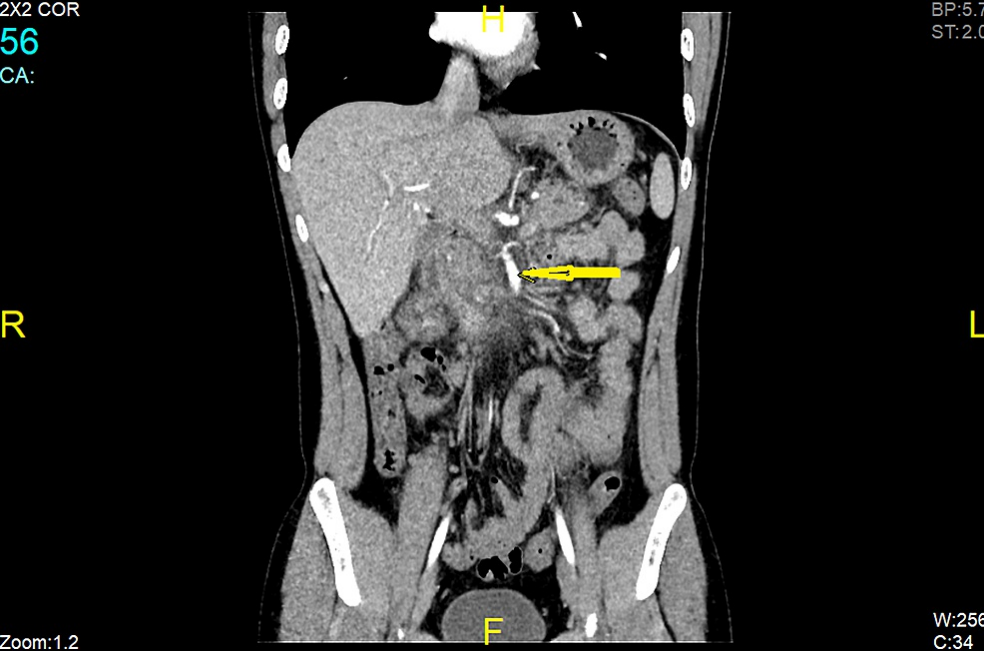
Coronal view of patent superior mesenteric artery lying adjacent to the area of the superior mesenteric vein thrombosis

An initial CT imaging revealed peri-pancreatic and parenchymal edema of the pancreas, consistent with AP. Also, moderate dilatation of the pancreatic duct without discrete lesion of the pancreatic head was observed. Aorta and Inferior vena cava were normal; however, the CT scan also showed a partially occluded and thrombosed superior mesenteric vein (see Figure 1 ) and superior mesenteric artery in Figure 2.

**Figure 3:**
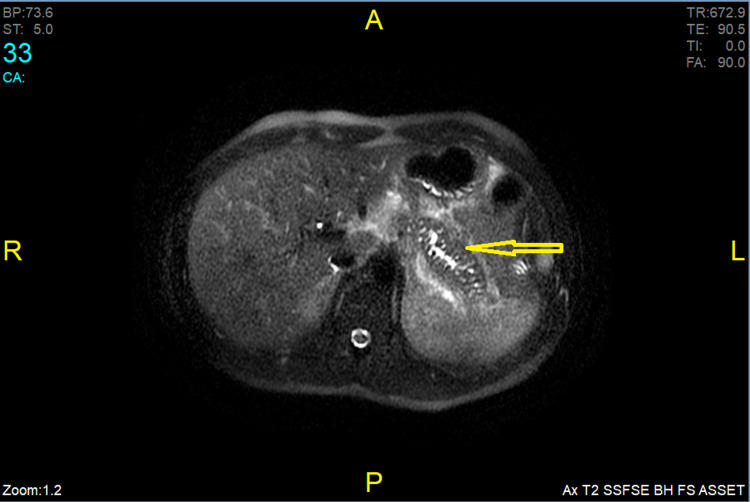
Arrow in the image showing edematous pancreas

Bowels, appendix, and colons were normal. A follow-up magnetic resonance cholangiopancreatography (MRCP) protocol of the abdomen was consistent with the CT scan findings of AP (see Figure 3).

Magnetic resonance imaging (MRI) of the abdomen with intravenous contrast (MRCP protocol) was initiated, and the results were consistent with the CT findings of AP. However, MRCP reported a focal area of dilation of duct or pseudo-cyst formation in the body of the pancreas measuring 8x8 mm.

Therapeutic dose anticoagulant was then commenced and the patient reported significant improvement in his symptoms by the third day of admission. After several weeks of follow-up in our outpatient clinic, the patient reports no abdominal pain and is still on a therapeutic anticoagulant (rivaroxaban).

## Discussion

Acute recurrent pancreatitis (ARP), a disease characterized by recurrent episodes of pancreatitis in a normo-functional gland, is usually suspected after the second episode of AP [3,4]. Among other etiologies of ARP, alcohol has been implicated as a common etiology [3,5,6]. This patient has had a history of recurrent alcohol use (10 glasses of vodka), frequently preceding his recurrent episodes of pancreatitis. Alcohol-induced ARP is thought to be dose-dependent (4-7 drinks per day in men and 3 or more drinks per day in women). However, less than 10% of people who frequently consume alcohol develop AP, which suggests that additional triggers or other factors are needed for pancreatitis to develop [6]. Even though the exact mechanism of pancreatic injury is not precise [5], many possible explanations have been studied and documented. In addition to other oxidative cells in the body, alcohol has been known to be oxidized in the cytosol of the pancreatic acini, activating a cascade of metabolic pathways that leads to oxidative and non-oxidative dose-dependent injury to the pancreas, including free radical stress and acinar damage [5,7,8]. CEL gene polymorphism can also modulate this etiopathogenesis, especially an increase in the L-allele frequency, cationic trypsinogen gene, and pancreatic secretory trypsin inhibitor (SPINK-1) [5,9]. Various other mechanisms of the etiology of pancreatic pain have been postulated to include sphincter of Oddi spasm, the ductal plug hypothesis with a background of ethanol-induced pancreatic enzymes effects like amylase and cholecystokinin, the alcohol-mediated endotoxin-induced pancreatic necrosis, and the ethanol-induced impaired blood flow to the pancreases which causes hypoxia [5,9,10]. This damage in pancreatic function is then modulated through various neurohormonal mechanisms to present as pain.

Abdominal pain, reported as one of the most common clinical presentations in pancreatitis, can present in various forms - sharp or dull - and is often described in the patients' best words. This pain has been explained to be due to complex neuropathologic and physiologic interplay. A special group of receptors in the afferent pancreatic neuronal pathway, called the silent nociceptors, are activated during inflammation of the pancreas and convey pain stimuli through vagal afferent to the brain [10,11]. These nociceptors are thought to be stimulated by various chemo- and mechano-sensing mechanisms (ischemia, stretching, and necrosis) that are yet to be fully understood.

These painful stimuli characteristically described to occur in the upper abdominal regions, often radiating to the back, have been classically documented in various clinical pancreatitis case reports [2,12-15]. It is thought that the complexity of the anatomical localization of pancreatic pain is partially related to a complex terminal field of intra-abdominal organs and regions innervated by the vagus nerve.

SMVT, a condition mediated by endothelia injury, inflammation, hypercoagulability, and stasis, is an entity that may exist independently, and have a causal or prognostic relationship with pancreatitis [16,17]. SMVT could present with nonspecific symptoms or pain [16]. The abdominal pain evoked in SMVT has significant overlap because AP and SMVT also present most commonly with abdominal pain (91-100% of cases) [16-18]. SMVT-associated pain is caused by reduced blood supply, leading to demand ischemia and necrosis resulting from thrombotic occlusion to the distal visceral supply [16]. The severity of pain presents various intensities depending on the rapidity of thrombus formation, the extent and location of vessel involvement, the degree of the inflammatory response, and the patient pain perception index [16,19].

Incidence of pancreatitis with SMVT and etiopathogenesis of the association

In various independent multicenter studies, the co-existence of SMVT and recurrent AP has been reported [12-14]. This symbiotic association has been thought to induce a procoagulatory state triggered by Intra-abdominal inflammation associated with AP [13-15]. This pancreatic inflammation-mediated coagulation disruption may also explain the Grey turner and Cullen sign seen in acute hemorrhagic pancreatitis [20].

This similarity in pain presentation may pose a diagnostic dilemma. Whereas no sensitive or specific marker is diagnostic for SMVT, serum lipase levels are most specific and sensitive for diagnosing AP [21] combined with other biologic markers - trypsinogen, c-reactive protein, procalcitonin, phospholipase A2, Interleukin 6, and Interleukin 8 that have limited clinical availability. However, high levels of metabolic acidosis and lactate dehydrogenase correlate with increased mortality [16]. Also, according to the 2013 ACG guideline, because AP can usually be diagnosed based on clinical symptoms and laboratory testing, contrast-enhanced CT scanning and/or MRI of the pancreas should be performed only in the absence of clinical improvement or if the diagnosis is unclear [22,23]. On the other hand, contrast-enhanced CT of the abdomen is a diagnostic modality for SMVT [16,24]. The characteristic finding for mesenteric thrombosis is a filling defect within a mesenteric vein, as depicted in Figures 1, 2. CT scan may include other nonspecific findings, like a thickened mesentery, indistinct bowel margins, bowel wall thickening, and ascites. Imaging should consist of the entire abdomen, with contrast timing for arterial and venous phases [16]. Abdominal CT scan confers sensitivity and specificity of 93% and 100%, respectively, with positive and negative predictive values between 94% and 100% [16,25]. A CT scan was subsequently performed in our patient that revealed the underlying associated SMVT, which was promptly managed by appropriate anticoagulation therapy.

Bowel rest with early resumption of enteral nutrition, intravenous fluid, and pain management are the goals of treatment for both AP and, if coexisting with SMVT, the addition of anticoagulants or in some cases thrombolysis are recommended to prevent infarction due to hypoperfusion [3,6,16,26]. While the American Gastroenterology Association suggests against the use of antibiotics in AP, prophylactic antibiotics may be useful in bowel ischemia [3,6,16,26]. Additionally, management with systemic anticoagulation in SMVT expedites recanalization and bowel reperfusion, prevents thrombus propagation, and decreases associated morbidity and mortality. Direct thrombolysis, surgery, and thrombectomy may also be useful in other clinical scenarios [16]. Our patient received low-molecular-weight heparin in the hospitalization period and was followed up as an outpatient. The recommended long-term management length is about six months on an anticoagulant for provoked cases and more than six months for most unprovoked cases of venous thrombosis [16]. The abdominal pain that was out of proportion despite adequate pain control resolved following initiation of the anticoagulant. Perhaps the resolution of the pain was the usual sequence of resolution for AP pain, or it may have been as a result of the resolution of the thrombus. The relationship in this sequence will be difficult to tell. Could this be a cause or course?

## Conclusions

This case highlights a rare, interesting relationship between two disease entities that could be a cause and/or effect of the other. The occurrence of SMVT and AP poses a diagnostic and management challenge and should be considered in the evaluation of a patient with abdominal pain. The patient was started on low-molecular-weight heparin and bridged to long-term anticoagulation - rivaroxaban 15 mg by mouth 2 times daily for 21 days, then 20 mg daily for 9 days, and discharged home after resolution of his pain, with a plan for anticoagulant continuation on outpatient follow-up. A subsequent follow-up CT scan did not show an SMVT. His SMVT was most likely secondary to inflammation from his pancreatitis or maybe an undiagnosed genetic hypercoagulability state. He is currently receiving anticoagulants and follow-up in our outpatient clinic. Fatal complications of AP should not be missed. Although a contrast-enhanced CT scan is not recommended in the early stages of diagnosis of AP in the ACG guideline, fatal complications such as SMVT can be avoided from being missed if a CT scan is deployed early during management.
